# Multiple origins of the apple seed microbiome: disentangling sexual and asexual transmission pathways

**DOI:** 10.1186/s40793-026-00901-y

**Published:** 2026-04-22

**Authors:** Maria Faticov, Ayco J. M. Tack, Doris Köberl, Gabriele Berg, Ahmed Abdelfattah

**Affiliations:** 1 Department of Wildlife, Fish and Environmental Studies, SLU, Umeå, Sweden; 2 Department of Physical Geography, SU, Stockholm, Sweden; 3 Leibniz Institute for Agricultural Engineering and Bioeconomy (ATB), Potsdam, Germany; 4 Institute for Biochemistry and Biology, University of Potsdam, Potsdam, Brandenburg Germany; 5 Institute of Environmental Biotechnology, Graz University of Technology, Graz, Styria Austria; 6 Department of Ecology , Environment and Plant Sciences, SU, Stockholm, Sweden

**Keywords:** Bacteria, Community assembly, Flower, Microbial transmission, Seed microbiome

## Abstract

**Background:**

The seed microbiome plays a key role in the assembly of the plant microbiome, which has major impacts on plant health. Nonetheless, little is known about the origin of the seed microbiome. We investigated the relative contributions of two potential transmission routes: sexual inheritance (via reproductive organs) and asexual inheritance (via the plant vascular system). To do that, we sampled flower ovaries and pollen sacs, fruiting spurs both before bloom and at seed maturity stages and mature seeds from five field-grown apple trees (*Malus domestica* Borkh. cv ‘Gala Galaxy Selecta’).

**Results:**

We showed that bacterial alpha diversity differed among tissues: spurs sampled before bloom had significantly higher richness and Shannon diversity than all other compartments, whereas ovary, pollen, spurs at seed maturity, and seeds did not differ in either richness or Shannon diversity. In addition, bacterial community composition differed significantly across all tissue types (ovary, pollen, spurs before bloom, spurs at seed maturity, and seeds). Source tracking revealed that both sexual (30.3%) and asexual (23.8%) pathways contributed to seed microbiome assembly, with spurs at seed maturity being the dominant source. Notably, a large proportion (45.9%) of the seed microbiome originated from unknown sources.

**Conclusions:**

Overall, such insights into seed microbiome origin offer new opportunities to enhance seed health and crop productivity through microbiome-assisted breeding.

**Supplementary Information:**

The online version contains supplementary material available at 10.1186/s40793-026-00901-y.

## Background

Seed-associated microbial communities can influence early plant development and may contribute to the assembly of the plant microbiome, with potential consequences for plant health and performance. Yet, the origin of the seed microbiome remains poorly understood, particularly the relative roles of (i) vertical transmission from parental tissues and (ii) horizontal acquisition from environmental reservoirs such as air, soil, water, and pollinators [1, 2]. Vertical transmission (often referred to as microbial inheritance) can carry microorganisms from parent plants into seeds and potentially maintain some microbial lineages across generations [3–5]. However, empirical evidence suggests that seed microbiomes can be either inherited or acquired from the environment and the balance between these sources likely varies across host species, plant organs, and developmental stages [5, 6]. Quantifying these contributions is therefore essential for understanding how the seed microbiome is assembled and which plant organs and environmental sources act as the most important reservoirs for seed microbes. More broadly, while inheritance has been proposed as one potential contributor to host–microbiome congruence across evolution (i.e., phylosymbiosis; [7]), testing such evolutionary patterns requires first establishing via which pathways microbes are transmitted to seeds.

The transmission of microbes from plant to seed can occur via two main pathways: sexual, involving reproductive organs such as pollen and ovaries [8, 9] and asexual, originating from vegetative tissues like shoots and fruiting spurs [5, 6]. For example, if the seed microbiome is predominantly originated from the sexual transmission pathway, one can expect that the microbial communities in spurs before bloom would also be important sources of microbes [10]. This is because floral organs develop directly from these spurs, and at the time of flowering, spur endophytes are likely the primary microbial source for emerging reproductive tissues [5, 11]. In other words, any microbes transmitted through pollen or ovary most likely originate from or pass through vegetative tissues. Conversely, if the seed microbiome is primarily shaped by asexual transmission, then fruiting spurs collected during seed maturation would be more important sources of the seed microbiome, as they remain connected through vascular system to the maturing seed and can enable microbial transfer through maternal tissues during seed filling. These two scenarios are not mutually exclusive, but each pathway implies distinct temporal and spatial patterns of microbial transmission toward the seed. In summary, sexual and asexual pathways are expected to lead to different microbial community structure across vegetative, floral, and seed tissues, because each pathway encompasses different microbial source pools, vascular and tissue connectivity, and duration of microbial exposure during the development. Understanding the relative contributions of sexual and asexual transmission pathways will not only clarify the mechanisms underlying microbial inheritance but also provide insights into the ecological and evolutionary forces shaping seed microbiomes.

Microbial communities are not homogeneously distributed within plants. Instead, different tissues such as stems, leaves, flowers, and reproductive organs tend to harbor distinct microbiomes shaped by unique physiological characteristics [12, 13]. Studies have shown that pollen grains can carry diverse microorganisms that could be transmitted during fertilization [14–16]. Paternal breeding lines contributed substantially to the seed microbiota of oilseed rape [17]. Although the presence of microorganisms in the plant ovules was reported [18] to this date much less is known about female than male reproductive parts [9]. Asexual tissues, such as shoots and stems, are typically longer-lived and repeatedly exposed to airborne and soil inoculum and insect contact, which can increase opportunities for microbial colonization relative to short-lived reproductive organs. Thus, asexual tissues can harbor higher diversity of microbes than sexual tissues. Interestingly, transmission pathways may differ among microbial taxa, with certain genera associated with specific tissues or transmission route [19]. Altogether it is important to consider both tissue-specific microbes and taxon-specific transmission strategies when studying plant-to-seed microbial inheritance.

**Fig. 1:**
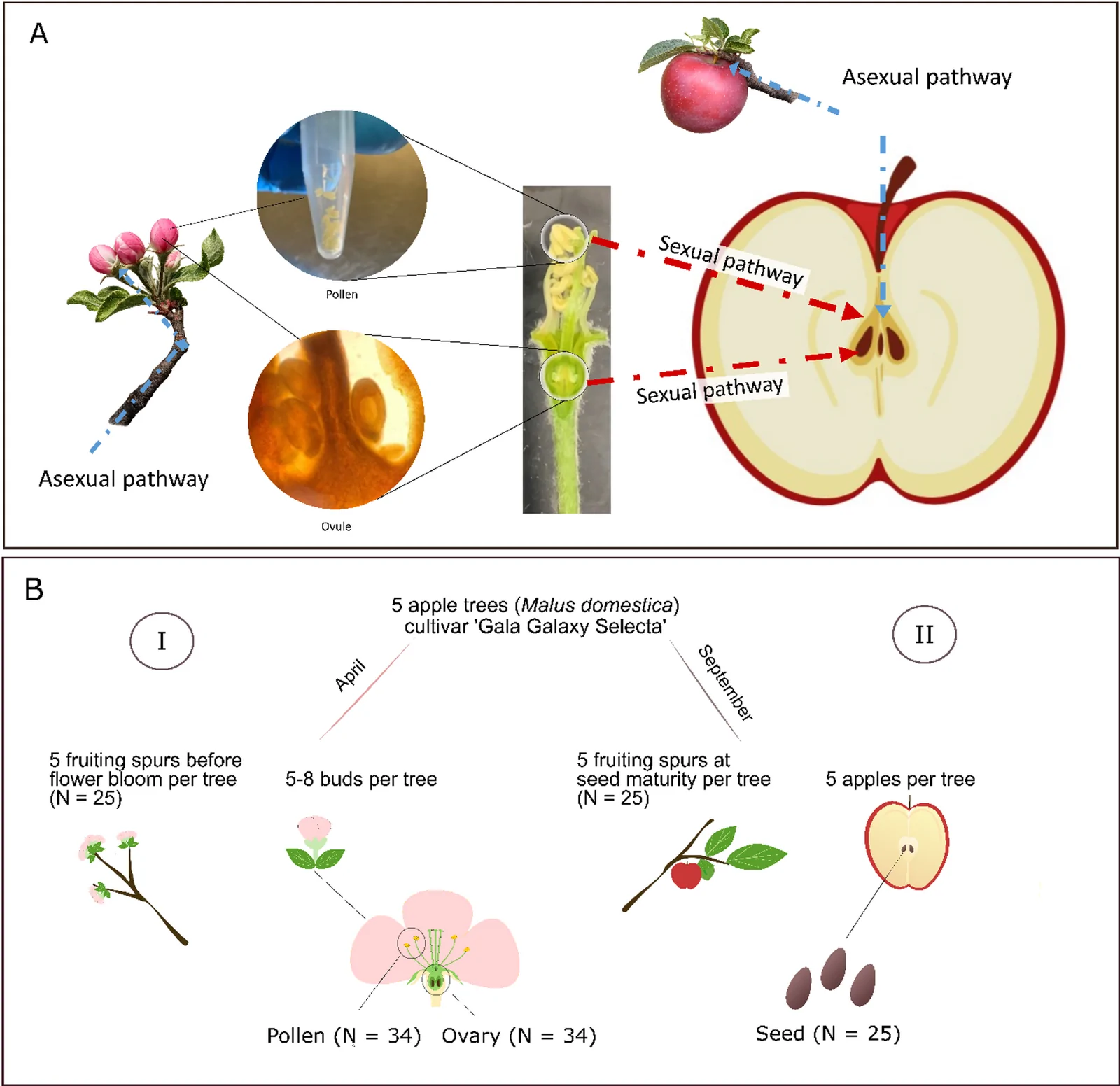
Schematic representation of sexual and asexual transmission pathways to the seed in apple trees. **A** Dashed orange arrows indicate the sexual transmission pathway, where microbes are transferred via floral reproductive tissues (pollen and ovary). Dashed blue arrows indicate the asexual transmission pathway, where microbes are transferred via fruiting spur tissue, sampled before bloom and at seed maturity. **B** Experimental design showing the sampling scheme. From each of five ‘Gala Galaxy Selecta’ apple trees, we collected samples from five spurs (I) before bloom and (II) at seed maturity. From the five trees, we also collected 5 to 8 floral buds; in the laboratory, each flower bud was dissected to isolate ovary and pollen. We also collected five apples from each of five apple trees and their seeds

This study aims to address the critical knowledge gap regarding the origins of the seed microbiome. Specifically, we aim to disentangle the contributions of sexual (pollen sacs and ovaries) and asexual (spurs endophytes) pathways to apple seed microbiome origin and assembly (Fig. 1). We predict that:

1. Microbial diversity and composition differ between spurs, ovary and pollen, with spurs harboring a higher diversity of microorganisms than the ovary and pollen.
2. Both sexual (ovary and pollen) and asexual (spurs before bloom and at seed maturity) pathways are predicted to contribute to seed microbiome assembly, though their relative importance can vary. Thus, we expected that:
  - If sexual transmission dominates, ovaries and pollen can serve as the main source for microbes colonizing seeds, making ovaries and pollen sacs similar in their community composition to seed.
  - If asexual transmission dominates, we expect spurs at seed maturity to contribute the largest proportion to the seed microbiome, due to microbial transmission through the vascular system to developing seeds.
3. A notable proportion of the seed microbiome originates from unknown sources, possibly reflecting unsampled environmental reservoirs such as pollinators, soil or air.

## Methods

### Study system and sample collection

To study the sexual and asexual pathways of bacterial endophytes transmission in plants, we selected five apple trees (*Malus domestica*, cultivar ‘Gala Galaxy Selecta’) from the Botanical Garden of the University of Graz (47.0814° N, 15.4562° E). Sampling was conducted at two time points: before bloom (April 15, 2020) and at seed maturity (September 9, 2020). All samples were collected using sterile forceps and gloves, transported on ice and processed under sterile conditions in a laminar flow cabinet.

We sampled tissues representing two putative transmission routes to the seed microbiome. The sexual pathway was represented by floral reproductive tissues (pollen and ovary) collected from flower buds before bloom (Fig. 1a). The asexual pathway was represented by the fruiting spur (maternal vegetative woody tissue supporting the reproductive structures and measuring 1–3 cm long) that is physically connected to the flower cluster and to the developing fruit, referred to as “spur” (Fig. 1a and Fig. S1).

To investigate the contribution of the two pathways to seed bacterial endophyte transmission, five to eight flower buds from each of five trees were collected right before tree bloom in April (Fig. 1b). Alongside sexual organs, we collected the asexual tissue, the spur connected to the flower cluster, using sterile tools (Fig. 1b and Fig. S1). In September, five mature apples were harvested, and seeds were extracted under sterile lab conditions (Fig. 1b). Alongside seeds, we also sampled five fruiting spurs with the apple fruits (Fig. 1b). The final set of samples included 34 pollen, 34 ovary and 25 fruiting spurs before bloom, 25 fruiting spurs at seed maturity and 25 seeds (Fig. 1b).

### Preparation of ovary, pollen, spur and seed samples

Under sterile laboratory conditions, each flower bud was dissected to isolate the female (ovary) and male (pollen sac) reproductive organs (Fig. 1a). The outer ovary layer was removed, and the entire inner portion was processed as a single ovary sample. Closed pollen sacs (hereafter referred to as *pollen*) were isolated from the same buds, with all pollen sacs from a single bud pooled into a single sample. Each ovary sample weighed approximately 0.02 g, and each pollen sample 0.03 g. Samples were stored at − 20 °C until DNA extraction.

Spurs collected before bloom and during seed maturity were surface sterilized in a 4% Sodium Hypochlorite (NaOCl) solution then rinsed three times with autoclaved Ultraopure water, each rinse lasting three minutes. After sterilization, the bark of the spurs was removed using a scalpel, and the first and last centimeters of the spurs were discarded. The spurs were then cut into 1 cm pieces and transferred into sterile bags. Each spur sample collected before bloom weighed approximately 0.11 g, whereas spur samples collected at seed maturity had a weight of 0.59 g. To prepare the samples, 10 mL of 0.85% sodium chloride (NaCl) solution was added, and the tissue was homogenized using a sterilized mortar and pestle. The homogenate was transferred into two Eppendorf tubes and centrifuged at 16,000 g for 20 min at 4 °C. After discarding the supernatant, 2 mL of NaCl solution was added to each tube for a second centrifugation under the same conditions. Seeds were processed using the same protocol. For each seed sample, 8 seeds (mean weight of 0.43 g) were ground in 10 mL of 0.85% NaCl solution, followed by centrifugation steps. All resulting pellets (from seeds and spurs) were stored at − 20 °C until DNA extraction.

### Molecular methods and bioinformatics

DNA extraction was performed using the FastDNA™ SPIN Kit for Soil (MP Biomedicals). DNA extractions were carried out separately for each sample type (ovules, pollen, seeds, spurs before bloom and at seed maturity) to prevent microbial cross-contamination, particularly between pollen and ovule samples. The 16 S rRNA gene was amplified using primers 515f and 806r [20]. To minimize host mitochondrial and plastid 16 S rDNA amplification, Peptide Nucleic Acid (PNA) clamps were added to the PCR mixture [21, 22]. PCR reactions were performed in a total volume of 30 µL, indexed and were purified using the Wizard SV Gel and PCR Clean-Up System (Promega, Madison, WI, USA; see Supporting Information for details). We included negative controls (no-template controls) for each of PCR runs and sequenced them together with samples. DNA concentration was measured with a Nanodrop 2000 spectrophotometer (Thermo Fisher Scientific, Wilmington, DE, USA), samples were pooled at equimolar concentrations and sequenced using 250 bp paired-end Illumina MiSeq sequencer.

Raw sequence data were processed using QIIME2 (version 2021.2) [23, 24]. Following paired-end read joining and quality filtering with Phred-score 33, error correction, denoising, chimera removal, and Amplicon Sequence Variant (ASV) table generation were performed using DADA2 [25]. Taxonomic assignment of ASVs was conducted using the SILVA reference database (version 132, 99% identity, 06.05.21), with VSEARCH implemented for de novo clustering. Sequences of host mitochondrial and chloroplast origin were filtered out. We identified potential contaminants in negative control using function *isContaminant* in *decontam* package (method = prevalence, threshold = 0.5) package in R. This analysis flagged five ASVs (assigned to the genera *Delftia*, *Brevundimonas*, *Lactobacillus*,* Devosia* and *Methylobacterium*) as contaminants, i.e., taxa with higher prevalence in negative controls than in biological samples. These ASVs accounted for 13,810 reads (0.19% of all reads in biological samples) and were removed prior to downstream analyses. Negative-control samples were then excluded from all downstream analyses and figures (see Supporting Information for details). After quality filtering and contaminant removal, the dataset contained 143 samples, 3237 ASVs and 7,380,768 sequences across biological samples, with sequencing depths ranging from 55 to 951,775 reads per sample. The pre-rarefaction ASV table contained on average 60 ASVs per sample (range: 3–252).

To assess whether alpha-diversity inferences were sensitive to rarefaction depth, we performed a sensitivity analysis by rarefying samples to 100, 200, 300, 500, 800, and 1,000 reads per sample (without replacement; fixed random seed), recalculating observed richness and Shannon diversity, and repeating tissue-level comparisons at each depth. We selected 500 reads for the main analyses as a compromise between sample retention and coverage; rarefaction curves and the model outcomes across depths are reported in Supporting Information (Fig. S2; Table S1). After rarefaction to 500 reads, we retained 124 samples (*N*_*ovary*_ = 25, *N*_*pollen*_ = 30, *N*_*spurBB*_ = 19, *N*_*spurSM*_ = 25 and *N*_*seed*_ = 25), totaling 62,000 sequences and 1464 ASVs.

### Statistical analysis

All analyses were conducted using R (version 4.4.3) [26].

To investigate bacterial community composition, species richness (the number of observed ASVs per sample) and diversity (Shannon index), the ASV table was rarefied to 500 reads per sample (Fig. S1). Pairwise differences in observed richness and Shannon diversity, among tissues (ovaries, pollen, spurs, and seeds) were evaluated using Tukey’s post hoc tests.

Prior to multivariate community composition analysis (PERMANOVA), cumulative Sum Scaling (CSS) from the *MetagenomeSeq* package was applied to account for differences in sequencing depth [27]. In the next step, the CSS transformed ASV matrix was used for beta diversity calculation, using Bray-Curtis dissimilarity metrics [28, 29]. Multivariate microbial community composition was visualized through Principal Coordinates Analysis (PCoA) using *plot_ordination* function in the *ggplot2* package [30]. Statistical testing of beta diversity was performed using PERMANOVA with the *adonis2* function in the *vegan* package [31]. To assess whether microbial communities in spurs before bloom were more similar to those at seed maturity than to those in ovary or pollen tissues, we calculated Bray–Curtis dissimilarities between sample pairs from spur at bloom and each of the following: spur at seed maturity, ovary, and pollen. Distances were extracted from the dissimilarity matrix and grouped by tissue pair. We then performed pairwise Wilcoxon rank-sum tests to compare the distributions of these between-group distances.

To explore the role of sexual (ovaries and pollen) and asexual (spur) tissues in seed microbiome transmission and assembly, we applied two complementary source-tracking approaches. First, we used a Bayesian source-tracking method implemented in SourceTracker v2.0.1 [32], which identifies taxa that differ among sources and sinks. For SourceTracker, we first collapsed samples by tissue type (ovary, pollen, spur before bloom, spur at seed maturity), and then rarefied both source and sink count tables to 1000 reads per tissue type as part of the standard model setup to reduce bias from uneven sequencing depth. Second, we applied FEAST v0.1.0 [33], an expectation–maximization framework that infers mixture proportions directly from integer ASV count data; FEAST was run for up to 1,000 iterations, using the same sources (ovary, pollen, spur before bloom, spur at seed maturity) and with the input count table rarefied to 1000 reads tissues type for comparability with SourceTracker. In both approaches, seeds were treated as “sink” samples, while ovary, pollen, and spur tissues were treated as “sources”. Both methods estimate an “unknown” fraction, representing ASVs detected in seed samples that are not explained by the sampled sources (i.e., potential contributions from unsampled reservoirs).

## Results

**Fig. 2:**
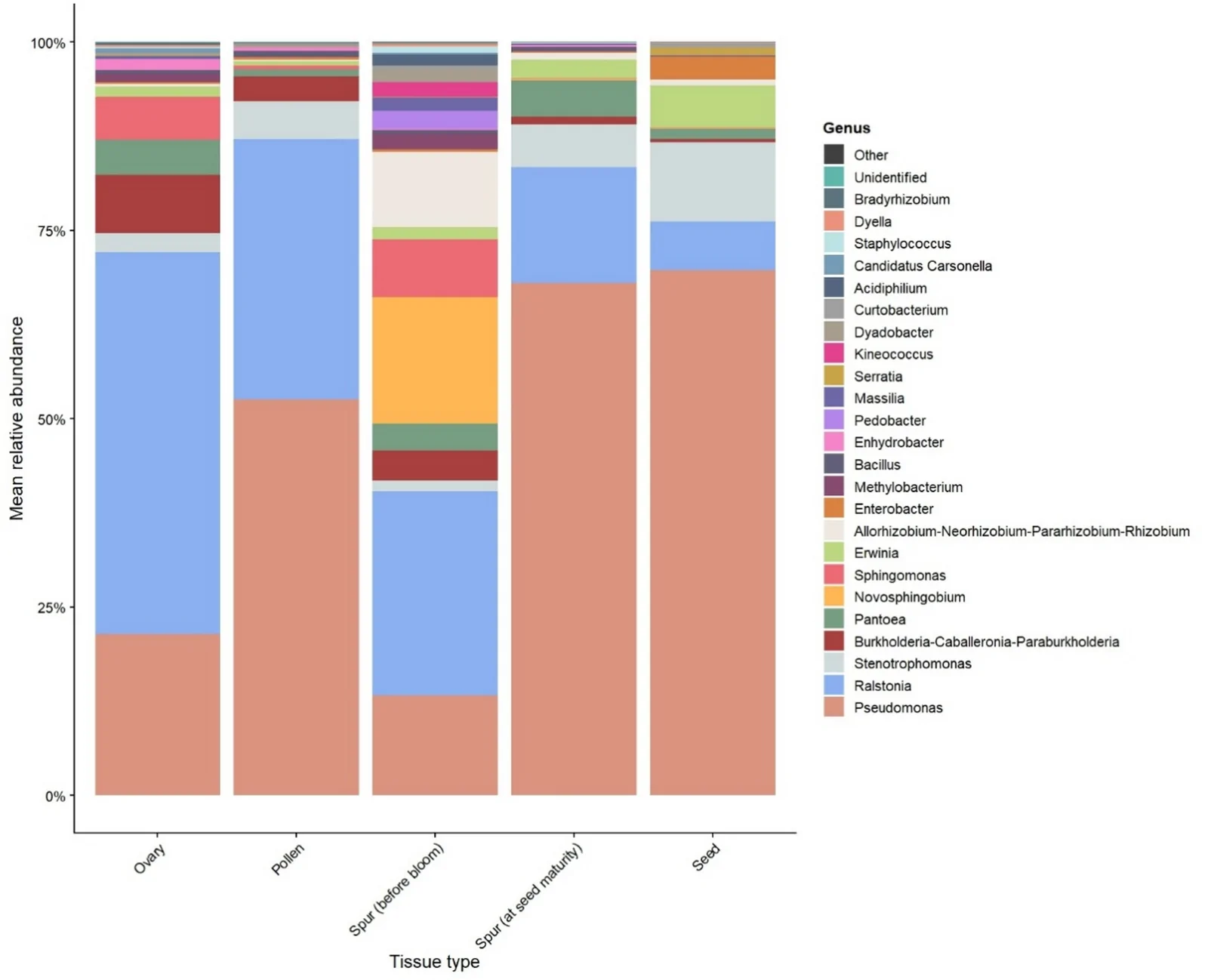
Relative abundance of bacterial genera in ovary, pollen, spurs before bloom and spurs at seed maturity of the apple trees (*Malus domestica* BORKH. cv ‘Gala Galaxy Selecta’). Bars represent the mean relative abundance across samples within each tissue. Shown are the 24 most abundant genera across the full dataset plus ‘Unidentified’ (ASVs lacking genus-level assignment); all remaining genera were pooled into ‘Other’. For the relative abundance of bacterial genus at the sample level see Fig. S3

The bacterial taxa with the highest relative abundance varied across tissues (Fig. 2; Fig. S3). In ovaries, the dominant genera were *Ralstonia* and *Pseudomonas*, followed by *Burkholderia*, *Pantoea* and *Sphingomonas* (Fig. 2). In pollen, the community was dominated by *Pseudomonas*, followed by *Ralstonia*, *Stenotrophomonas* and *Burkholderia* among other genera (Fig. 2). For spurs, the composition shifted with developmental stage: before bloom, *Ralstonia*, *Novosphingobium* and *Pseudomonas* were the most prevalent genera, but several other taxa were also abundant (e.g. *Sphingomonas*), whereas at seed maturity, *Pseudomonas* was the most relatively abundant, followed by *Ralstonia* (Fig. 2). Finally, in seeds, *Pseudomonas* was the most abundant genus, followed by *Stenotrophomonas*, *Ralstonia* and *Erwinia*. Interestingly, *Erwinia* was also present in ovaries and spurs at seed maturity (Fig. 2).

### The richness, diversity and composition of the spur, ovary, pollen and seed microbiomes

**Fig. 3:**
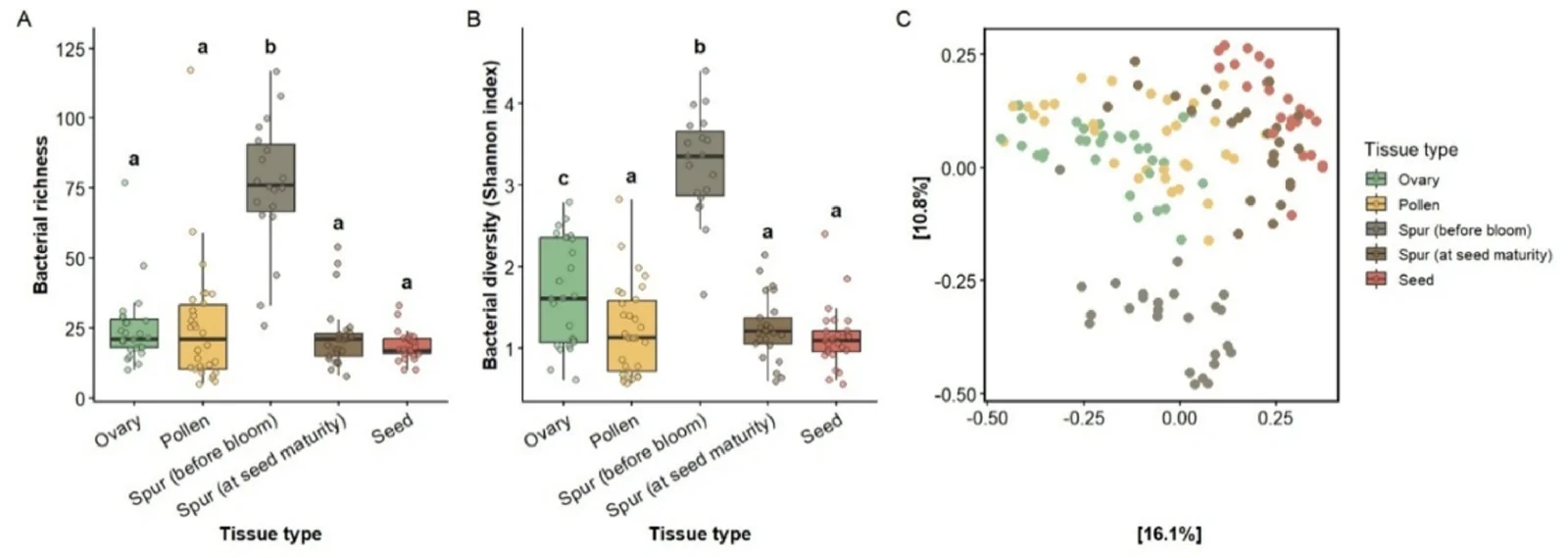
The differences in bacterial observed richness (**a**), Shannon diversity (**b**) and community composition (**c**) among ovary, pollen, spurs before bloom, spurs at seed maturity and mature seed of the apple trees (*Malus domestica* BORKH. cv ‘Gala Galaxy Selecta’). **a**–**b** Differences in observed species richness and Shannon diversity among tissue types. The horizontal line within each box represents the median, the box edges show the interquartile range (IQR), and the whiskers demonstrate the data range. The small circles represent raw data points, which are horizontally jittered to avoid overlap. **c** Difference in community composition among tissue types visualized with two-dimensional principal coordinates analysis (PCoA) of bacterial communities based on Bray–Curtis dissimilarity index.

Bacterial richness differed significantly among tissue types (one-way ANOVA: F_4,119_ = 42.55, *p* < 0.0001; Fig. 3a). Richness was highest in spurs before bloom, whereas ovary, pollen, spurs at seed maturity, and seeds had lower richness. Post-hoc comparisons showed that spurs before bloom harbored significantly higher richness than all other tissues (Table S2a), while no significant differences were detected among ovary, pollen, spurs at seed maturity, and seeds (Fig. 3a; Table S2a). Shannon diversity also varied significantly across tissue types (F_4,119_ = 56.90, *p* < 0.0001; Fig. 3b). Spurs before bloom showed higher diversity than all other tissues (Table S2b). In contrast, pollen, spurs at seed maturity, and seeds did not differ significantly from one another, whereas ovary had slightly higher diversity than pollen, spurs at seed maturity, and seeds (Fig. 3b; Table S2b).

Bacterial community composition varied significantly across tissue types (R^2^ = 0.14, F = 5.30, *p* = 0.001; Fig. 3c), with all pairwise comparisons showing a significant differences (Table S3). The most pronounced compositional difference was observed between spur endophytes before bloom and seeds (R^2^ = 0.17) (Fig. 3c; Table S3). While spur tissues also differed significantly from each other (R^2^ = 0.15), their microbial community composition were more similar to ech other than to those in ovaries (Wilcoxon rank-sum test, *p* = 0.045), but not significantly more similar than to those of pollen (Wilcoxon rank-sum test, *p* = 0.128).

### Sources of the seed microbiome

**Fig. 4:**
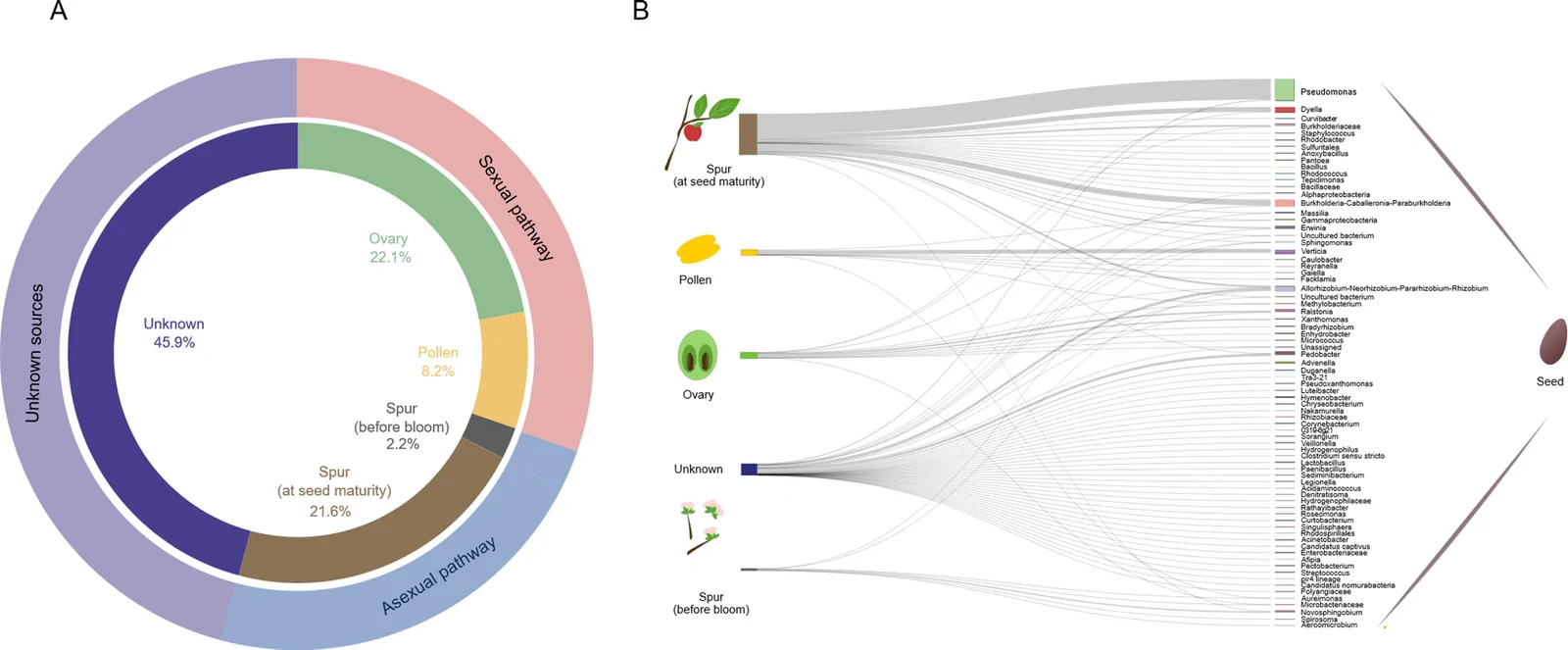
Identification of potential asexual and sexual pathways of microbiome transmission to apple seeds. **A** The inner donut chart shows the relative contribution of ovary, pollen and spur endophytes to the seed microbiome, as estimated using fast expectation-maximization for microbial source tracking (SourceTracker). The outer donut shows the sexual pathway represented by reproductive tissues (ovary and pollen) and the asexual pathway represented by maternal vegetative tissues (fruiting spurs); contributions from unsampled reservoirs are grouped as ‘Unknown’. For corresponding standard deviations (SD) and variation between individual trees see Fig. S3. **B** Relative contribution of different sources to the individual genera of the seed microbiome, as estimated using SourceTracker. Each line represents a taxon, with the thickness of the line corresponding to the relative contribution of a given source to the abundance in the seed microbiome. The thickness of each bar on the right shows the overall relative abundance of that genus in the seed microbiome. Taxa are shown at the genus level.

Both sexual (ovaries and pollen) and asexual (spurs at bloom and at seed maturity) sources made substantial contributions to the seed microbiome, accounting for 30.3% and 23.8%, respectively (Fig. 4a). From the sexual sources, the ovary (22.1%) contributed more than pollen (8.2%; Fig. 4a). From the asexual sources, spurs endophytes at seed maturity contributed a much large fraction of microorganisms to the seed microbiome (21.6%) than the spur endophytes at bloom (2.2%; Fig. 4a). Unknown sources accounted for 45.9% of the seed microbiome. Source tracking at the individual tree level showed among-tree variation in source contribution to the seed microbiome (Fig. S4). FEAST analyses showed qualitatively similar patterns (Figs. S5–S6), with pollen (32%) and spurs at seed maturity (35%) contributing most to the seed microbiome, whereas ovary (5%) and spurs before bloom (1%) contributed only marginally. Unknown sources accounted for 28% of the seed microbiome (Figs. S5–S6).

SourceTracker analysis also revealed taxon-specific transmission patterns to seeds (Fig. 4b; Table S4). Several dominant genera were primarily attributed to spurs at seed maturity, including *Pseudomonas* and *Burkholderia*. For *Pseudomonas* smaller contributions were also assigned to the ovary, for *Burkholderia* to the ovary and pollen (Table S4). Other taxa showed mixed origins, for example *Dyella*, the fire blight genus *Erwinia*, which were attributed to spurs at seed maturity, pollen and “Unknown” sources (Fig. 4b; Table S4). Bacteria from genus *Ralstonia*, for example, was attributed to the spurs at seed maturity, ovary and pollen (Fig. 4b; Table S4). In contrast, several genera were most strongly associated with reproductive tissues, including *Verticia* (pollen) and *Bradyrhizobium* (ovary and pollen) (Fig. 4b; Table S4). Finally, a subset of seed taxa was attributed predominantly to unknown sources, including *Pedobacter* and *Allorhizobium* (Fig. 4b; Table S4).

## Discussion

​ In this study we explored the role of sexual (pollen sacs and ovaries) and asexual (fruiting spurs) pathways to seed microbiome. We found that bacterial alpha diversity differed among tissue types, with spurs before bloom showing significantly higher richness and Shannon diversity than all other tissues, while ovary, pollen, spurs at seed maturity, and seeds were similar in species richness and Shannon diversity. We also found that community composition varied significantly among ovaries, pollen, shoots at bloom, shoots at seed maturity and seed. Sexual and asexual pathways contributed similar proportions to the seed microbiome, with spurs at seed maturity contributing the largest proportion, as followed by the ovary and pollen. We also observed pathway-specific associations for several bacterial taxa: Notably, nearly half of the seed-associated bacterial community could not be traced to the sampled tissues, suggesting that environmental reservoirs such as soil, air or pollinators likely play a substantial role in the origin of the seed microbiome. Overall, these findings indicate the importance of both sexual and asexual pathways in shaping seed bacterial endophytes.

Spurs before bloom harbored significantly higher bacterial richness and Shannon diversity than all other tissues, whereas spurs at seed maturity did not differ from ovary, pollen, or seeds for richness. Notably, species richness was higher in spurs before bloom than in spurs at seed maturity. A likely explanation is that spurs before bloom serve as an overwintering organ for many bacterial taxa that colonize spurs in the previous season. As tree growth resumes and season progresses, the spurs environment changes affected by higher plant defenses, which can negatively affect the number and diversity of bacterial taxa on fruiting spurs [34]. Bacterial community composition, on other hand, differed among all tissue types, with the strongest differences observed between spurs before bloom and seeds. Interestingly, although spur endophytes before bloom and at seed maturity were significantly different in bacterial community composition, they remained more similar to each other than to ovary communities, whereas spur communities were not significantly more similar to each other than to pollen. Pollen may serve as microhabitat that support a diverse microbial community due to their high nutrient content, such as sugars, lipids, and proteins [35, 36]. Interestingly, even though ovary had lower bacterial richness than spurs before bloom, they contributed a similar proportion of the seed microbiome. This pattern suggests that high bacterial alpha diversity in a source tissue does not necessarily translate into higher microbial transmission to the seed; instead, transmission may be shaped by developmental “opportunity window”. One avenue for future research is to examine when and how microbes disperse between tissues during plant development.

We expected that if sexual pathway played a greater role in microbial transmission, microbes from fruiting spurs before bloom might colonize floral organs (ovary and pollen) during their formation, which would later serve as sources for seed microbial communities. This is plausible given that floral tissues originate from vegetative shoots and can share endophytic communities. Alternatively, if asexual transmission dominated, spurs at seed maturity would contribute more to the seed microbiome via vascular connections that facilitate microbial dispersal to the developing seeds. Our findings supported the prediction that sexual and asexual pathways can contribute nearly equally to microbial transmission to the seed. Sexual tissues together contributed ~ 30%, with the ovary contributing more (22%) than pollen (8%), though both were consistent sources across tree individuals. The contribution of both male and female reproductive tissues to the seed microbiome may increase microbial diversity and functional redundancy in seeds, particularly under cross-fertilization scenarios [5]. In parallel, fruiting spurs endophytes at seed maturity were the most dominant contributors (~ 21.6%), indicating that transmission may occur during later stages of seed development. This is biologically plausible and supported by previous studies [11, 37], that suggest that as seeds mature alongside the fruit, vascular connections between maternal tissues and the developing seed are likely the strongest.

To date, very few published studies have explored the transmission of microbial communities between plant organs (e.g., ovary, pollen and fruiting spurs) and seeds, so the range of bacterial taxa that may colonize seeds via sexual and asexual transmissions is largely unknown. However, a few studies that explored microbial inheritance using watermelons and oaks [9] reported similar results for some of the taxa. For example, showing that, e.g. *Ralstonia* and *Bradirhizobium* are likely to be transmitted from the flower to the seed of the plants. As for other taxa, *Pseudomonas*, a dominant seed-associated genus, was primarily traced back to spur endophytes at seed maturity, with a smaller contribution from the ovary. This aligns with a meta-analysis across 50 plant species, which identified *Pseudomonas* as one of the most prevalent and abundant seed-associated bacteria [38]. Interestingly, *Pseudomonas* has also been shown to transmit from seeds to the leaves and roots of oak seedlings [6], suggesting its central role in both vertical inheritance and subsequent colonization of vegetative tissue. Among common pathogens of apple trees, *Erwinia*, a causal agent of fire blight disease, was shown to be present in seeds and to be mainly transmitted to seeds from spurs at seed maturity and unknown sources and too much lesser extent from pollen. Several studies have demonstrated the presence of *Erwinia* on stigmas of apple flowers with the possibility that pathogen overwinters on shoots [39, 40]. Several other abundant taxa, such as a Gram-negative genus *Pedobacter* and *Allorhizobium*, were also shown to be transmitted from unknown sources. *Pedobacter* has been mainly described from the soils [41], while *Allorhizobium* is primarily known as a nitrogen-fixing symbiont commonly associated with plant root nodules [42]. Their presence in apple seeds suggests potential, yet unidentified, transmission pathways or reservoirs within apple trees or surrounding environments, highlighting knowledge gaps in our understanding of microbial inheritance in plant microbiomes.

One interesting result is that nearly half of seed microbiota originated from parent tissues and half from unknown sources (e.g., Abdelfattah et al., 2023). Plausible sources include airborne deposition (aerosols/dust), soil-derived particles, and flower visitors/pollinators, as well as epiphytic floral surfaces and nectar. In fact, studies have shown that pollinators can act as vectors of plant-associated microbes, facilitating microbial exchange during flower visits and potentially contributing to seed microbiome assembly [15, 36, 43]. During foraging, pollinators come into contact with floral organs and nectar, carrying microorganisms on their body surfaces, mouthparts, or digestive tracts. Moreover, the floral microbiome itself is shaped by pollinator identity and behavior, which may influence the microbial communities on reproductive tissues [44–46]. Overall, future work that jointly explores potential microbial reservoirs including bioaerosols, soil, pollinator body surfaces and guts, and nectar epiphytes, alongside endophytic compartments across developmental stages will be essential to characterize all potential sources contributing to the seed microbiome.

## Conclusions

Our results also raise the question of whether the assembly follows stochastic or deterministic principles. First, if you follow the transmission pathways, it looks like a deterministic process, as all organs have very specific microbiomes. Second, the maternal microbiome changes during fruit ripening, which is also reflected in the seed microbiome, and even shows interesting parallels to the human microbiome [47]. Third, nearly half of the seed bacterial endophytes could not be traced to the sampled tissues and were attributed to unknown sources. Future studies can therefore focus on both endophytic and epiphytic communities across plant developmental stages (including floral and fruit surfaces), alongside soil, bioaerosols, and the role of pollinators in microbial transmission. Increased replication across tree individuals and tissue types, as well as standardized sampling by tissue mass and time point, will also be needed to quantify within-plant heterogeneity and strengthen the inference about transmission pathways. Overall, understanding the roles of sexual and sexual transmission routes has important applied implications for agriculture, as manipulating microbial inheritance could help enhance seedling health, for example disease resistance, through microbiome-assisted breeding.

## Supplementary Information

Below is the link to the electronic supplementary material.

Supplementary Material 1

## Data Availability

The data that supports the findings of this study, including the ASV table, metadata, R code, are archived in Zenodo repository. These resources can be accessed here via doi: 10.5281/zenodo.15639819 ( https://zenodo.org/records/15639819 ). All amplicon sequencing data generated in this study is deposited on the National Center for Biotechnology Information’s (NCBI) Sequence Read Archive under BioProject accession number PRJNA1308301. They can be accessed through the following link: https://www.ncbi.nlm.nih.gov/bioproject/1308301.
