# Supplementary material for: Multiple origins of the apple seed microbiome: disentangling sexual and asexual transmission pathways
Source: Environ Microbiome. 2026 Apr 22;21:59. doi: 10.1186/s40793-026-00901-y (PMC13126790; doi:10.1186/s40793-026-00901-y)
Supplement: Supplementary file 1 — Supplementary Material 1 [file 40793_2026_901_MOESM1_ESM.docx]

**Environmental Microbiome**

**Supplementary information**

Multiple origins of the apple seed microbiome: disentangling sexual and asexual transmission pathways

Maria Faticov^1,2^, Ayco J. M. Tack^2^, Doris Ortner^5^, Gabriele Berg^3,4,5#^, and Ahmed Abdelfattah^3, 5#*^

**Methods S1** Detailed description of molecular methods and bioinformatics

DNA was extracted using the FastDNA™ SPIN Kit for Soil (MP Biomedicals). Cell lysis was achieved using a FastPrep Instrument (MP Biomedicals) at 6.0 m/s for 40 seconds. We used 515f and 806r (Walters *et al.*, 2015) to amplify 16S. PCR reactions were run in volume of 30 µL, containing PCR-grade water, 6 µL Taq&Go (MP Biomedicals, Illkirch, France), 0.45 µL of each PNA, 1.2 µL of each primer, and 1 µL template DNA. The PCR protocol consisted of an initial denaturation at 95°C for 5 minutes, followed by 30 cycles of denaturation at 95°C for 30 seconds, a PNA annealing step at 78°C for 5 seconds, primer annealing at 54°C for 30 seconds, and extension at 72°C for 30 seconds, with a final extension at 72°C for 5 minutes. In total, we included five negative controls (blank PCR controls; negC1–negC5) that were processed and sequenced alongside the biological samples. The Wizard SV Gel and PCR Clean-Up System (Promega, Madison, WI, United States) was used to purify the PCR products. Concentration of the resulting DNA was measured with Nanodrop 2000 (Thermo Fisher Scientific, Wilmington, DE, United States), the samples were indexed and pooled in equimolar concentrations, and sequenced with 250 bp paired end Illumina MiSeq.

Raw sequence data were processed using QIIME2 (version 2021.2) (Caporaso *et al.*, 2010; Bolyen *et al.*, 2019). The raw read count was 11,334,276, after filtering and removing chimeric reads, the read count decreased to 7,394,578, corresponding to an overall retention of 65% of raw reads. The proportion of plant-assigned reads (chloroplast + mitochondria) differed across tissue types in the reprocessed dataset. For example, seeds had the highest sequencing depth of 3,165,390 total reads and the lowest plant fraction, whereas ovaries had 1,015,991 total reads but were dominated by plant reads. Specifically, the mean fraction of plant-assigned reads was highest in ovary samples (94.0%), followed by pollen (72.1%), spurs before bloom and at seed maturity (42.4%) and seeds (18.4%). As the next step, we identified potential contaminants in five negative controls using function *isContaminant* in *decontam* package (method = prevalence, threshold = 0.5) package in R. This analysis flagged five ASVs (assigned to the genera *Delftia*, *Brevundimonas*, *Lactobacillus,* *Devosia* and *Methylobacterium*) as contaminants, i.e., taxa with higher prevalence in negative controls than in biological samples. These ASVs accounted for 13,810 reads (0.19% of all reads in biological samples) and were removed prior to downstream analyses. After quality filtering and contaminant removal, the dataset contained 143 samples, 3,237 ASVs and 7,380,768 sequences across biological samples, with sequencing depths ranging from 55 to 951,775 reads per sample. The pre-rarefaction ASV table contained on average 60 ASVs per sample (range: 3-252).**Table S1.** Differences in bacterial observed richness and Shannon diversity across rarefaction depths.

| Rarefaction depth | n_samples | Alpha-diversity metric | df1 | df2 | F | P |
| --- | --- | --- | --- | --- | --- | --- |
| 100 | 140 | Observed richness | 4 | 135 | 38.35 | **<0.001** |
| 100 | 140 | Shannon diversity | 4 | 135 | 49.13 | **<0.001** |
| 200 | 135 | Observed richness | 4 | 130 | 36.27 | **<0.001** |
| 200 | 135 | Shannon diversity | 4 | 130 | 56.22 | **<0.001** |
| 300 | 131 | Observed richness | 4 | 126 | 40.79 | **<0.001** |
| 300 | 131 | Shannon diversity | 4 | 126 | 57.55 | **<0.001** |
| 500 | 124 | Observed richness | 4 | 119 | 42.55 | **<0.001** |
| 500 | 124 | Shannon diversity | 4 | 119 | 56.90 | **<0.001** |
| 800 | 116 | Observed richness | 4 | 111 | 30.76 | **<0.001** |
| 800 | 116 | Shannon diversity | 4 | 111 | 55.67 | **<0.001** |
| 1000 | 112 | Observed richness | 4 | 107 | 30.34 | **<0.001** |
| 1000 | 112 | Shannon diversity | 4 | 107 | 59.01 | **<0.001** |

**Table S2.** Pairwise comparisons of observed bacterial richness and Shannon diversity among different plant tissues: seed, ovary, pollen and spurs before bloom and at seed maturity. Estimates, standard errors (SE), degrees of freedom (df), t-ratios, and adjusted p-values (Tukey method) are shown. Significant comparisons (p < 0.05) are in bold.

|  | Estimate | SE | df | t-ratio | Adj. p-value |
| --- | --- | --- | --- | --- | --- |
| 1. Bacterial Observed richness |  |  |  |  |  |
| Ovary vs Pollen | -0.26 | 4.45 | 119 | -0.06 | 1 |
| Ovary vs Seed | 6.20 | 4.45 | 119 | 1.33 | 0.671 |
| Ovary vs Spur (Bloom) | -50.94 | 5.00 | 119 | -10.18 | **<0.001** |
| Ovary vs Spur (Seed maturity) | 2.92 | 4.65 | 119 | 0.63 | 0.970 |
| Pollen vs Seed | 6.47 | 4.45 | 119 | 1.45 | 0.595 |
| Pollen vs Spur (Bloom) | -50.67 | 4.82 | 119 | -10.52 | **<0.001** |
| Pollen vs Spur (Seed maturity) | 0.56 | 5.46 | 119 | 0.10 | 1 |
| Seed vs Spur (Bloom) | -50.67 | 4.81 | 119 | -11.42 | **<0.001** |
| Seed vs Spur (Seed maturity) | -3.28 | 4.65 | 119 | -0.70 | 0.955 |
| Spur (Bloom) vs Spur (Seed maturity) | 53.85 | 5.00 | 119 | 10.76 | **<0.001** |
| 1. Bacterial Shannon diversity |  |  |  |  |  |
| Ovary vs Pollen | 0.45 | 0.14 | 119 | 3.12 | **0.019** |
| Ovary vs Seed | 0.55 | 0.15 | 119 | 3.63 | **0.004** |
| Ovary vs Spur (Bloom) | -1.57 | 0.16 | 119 | -9.67 | **<0.001** |
| Ovary vs Spur (Seed maturity) | 0.44 | 0.15 | 119 | 2.92 | **0.034** |
| Pollen vs Seed | 0.45 | 0.14 | 119 | 0.68 | 0.961 |
| Pollen vs Spur (Bloom) | -2.02 | 0.16 | 119 | -12.92 | **<0.001** |
| Pollen vs Spur (Seed maturity) | 0.10 | 0.14 | 119 | -0.07 | 1 |
| Seed vs Spur (Bloom) | -2.12 | 0.16 | 119 | -13.05 | **<0.001** |
| Seed vs Spur (Seed maturity) | -0.11 | 0.15 | 119 | -0.72 | 0.952 |
| Spur (Bloom) vs Spur (Seed maturity) | 2.01 | 0.16 | 119 | 12.39 | **<0.001** |

**Table S3.** Pairwise comparisons of bacterial community composition among different plant tissues: seed, ovary, pollen and spurs before bloom and seed maturity. Degrees of freedom (df), Sum of Squares (SS), F-value (F), partial coefficient of determination (R²), p-value and adjusted p-value (Adj. p-value) are shown. Significant comparisons (p < 0.05) are in bold.

| Contrasts | df | SS | F | R² | p-value | Adj. p-value |
| --- | --- | --- | --- | --- | --- | --- |
| Ovary vs Pollen | 1 | 0.803 | 1.90 | 0.03 | 0.001 | **0.01** |
| Ovary vs Spur (Bloom) | 1 | 2.823 | 7.34 | 0.12 | 0.001 | **0.01** |
| Ovary vs Seed | 1 | 2.030 | 5.65 | 0.09 | 0.001 | **0.01** |
| Ovary vs Spur (Seed maturity) | 1 | 1.463 | 3.80 | 0.06 | 0.001 | **0.01** |
| Pollen vs Spur (Bloom) | 1 | 2.699 | 6.95 | 0.12 | 0.001 | **0.01** |
| Pollen vs Seed | 1 | 1.594 | 4.40 | 0.08 | 0.001 | **0.01** |
| Pollen vs Spur (Seed maturity) | 1 | 1.202 | 3.09 | 0.06 | 0.001 | **0.01** |
| Spur (Bloom) vs Seed | 1 | 3.157 | 10.12 | 0.17 | 0.001 | **0.01** |
| Spur (Bloom) vs Spur (Seed maturity) | 1 | 2.792 | 8.16 | 0.15 | 0.001 | **0.01** |
| Seed vs Spur (Seed maturity) | 1 | 1.193 | 3.81 | 0.07 | 0.001 | **0.01** |

**Table S4.** Taxon-specific source attributions to the seed microbiome calculated with SourceTracker. For each bacterial genus detected in seeds, the table reports the within-genus proportion of seed-associated abundance attributed by SourceTracker to each of four tissue types (ovary, pollen, spur before bloom, spur at seed maturity) and “Unknown” sources. Proportions sum to 1 within each genus.

| Bacterial genus | Prop. ovary | Prop. pollen | Prop. spur before bloom | Prop. spur at seed maturity | Prop. Unknown |
| --- | --- | --- | --- | --- | --- |
| *Pseudomonas* | 0.13 | 0.05 | 0.001 | 0.72 | 0.10 |
| *Burkholderia-Caballeronia-Paraburkholderia* | 0.28 | 0.31 | 0 | 0.41 | 0.01 |
| *Dyella* | 0.01 | 0.24 | 0.08 | 0.56 | 0.11 |
| *Allorhizobium-Neorhizobium-Pararhizobium-Rhizobium* | 0.01 | 0.01 | 0.04 | 0.40 | 0.55 |
| *Verticia* | 0 | 0.58 | 0 | 0.005 | 0.41 |
| *Unidentified/uncultured* | 0.13 | 0.27 | 0.05 | 0.36 | 0.19 |
| *Pedobacter* | 0.005 | 0.005 | 0 | 0.02 | 0.97 |
| *Erwinia* | 0 | 0.004 | 0 | 0.60 | 0.39 |
| *Ralstonia* | 0.44 | 0.14 | 0.02 | 0.38 | 0.02 |
| *Novosphingobium* | 0 | 0 | 0.94 | 0.05 | 0.01 |
| *Xanthomonas* | 0.69 | 0 | 0 | 0 | 0.31 |
| *Advenella* | 0 | 0 | 0 | 0.02 | 0.98 |
| *Massilia* | 0.10 | 0.02 | 0.08 | 0.69 | 0.12 |
| *Bradyrhizobium* | 0.51 | 0.37 | 0 | 0 | 0.12 |
| *Duganella* | 0.03 | 0 | 0 | 0 | 0.97 |
| *Anoxybacillus* | 0 | 0 | 0 | 1 | 0 |
| *Pseudoxanthomonas* | 0.025 | 0.025 | 0 | 0 | 0.95 |
| *Staphylococcus* | 0.10 | 0.30 | 0.02 | 0.56 | 0.02 |
| *Methylobacterium* | 0.46 | 0.09 | 0.34 | 0.06 | 0.04 |
| *Caulobacter* | 0.15 | 0.60 | 0 | 0.20 | 0.05 |
| *Hymenobacter* | 0 | 0 | 0 | 0 | 1 |
| *Chryseobacterium* | 0 | 0 | 0 | 0 | 1 |
| *Sulfuritalea* | 0.23 | 0.17 | 0 | 0.60 | 0 |
| *Enhydrobacter* | 0.57 | 0 | 0 | 0.43 | 0 |
| *Micrococcus* | 0.70 | 0 | 0 | 0 | 0.30 |
| *Rhodanobacter* | 0 | 0.10 | 0 | 0.90 | 0 |
| *Pectobacterium* | 0 | 0 | 0 | 0 | 1 |
| *Sphingomonas* | 0 | 0 | 0.13 | 0.59 | 0.28 |
| *Luteibacter* | 0 | 0 | 0 | 0 | 1 |
| *Pantoea* | 0 | 0 | 0 | 0.90 | 0.10 |
| *Rhodococcus* | 0.10 | 0.30 | 0 | 0.60 | 0 |
| *Reyranella* | 0.40 | 0.60 | 0 | 0 | 1 |
| *Curtobacterium* | 0 | 0 | 0 | 0 | 1 |
| *Hydrogenophilus* | 0 | 0 | 0 | 0 | 1 |
| *Rathayibacter* | 0 | 0 | 0.10 | 0 | 0.90 |


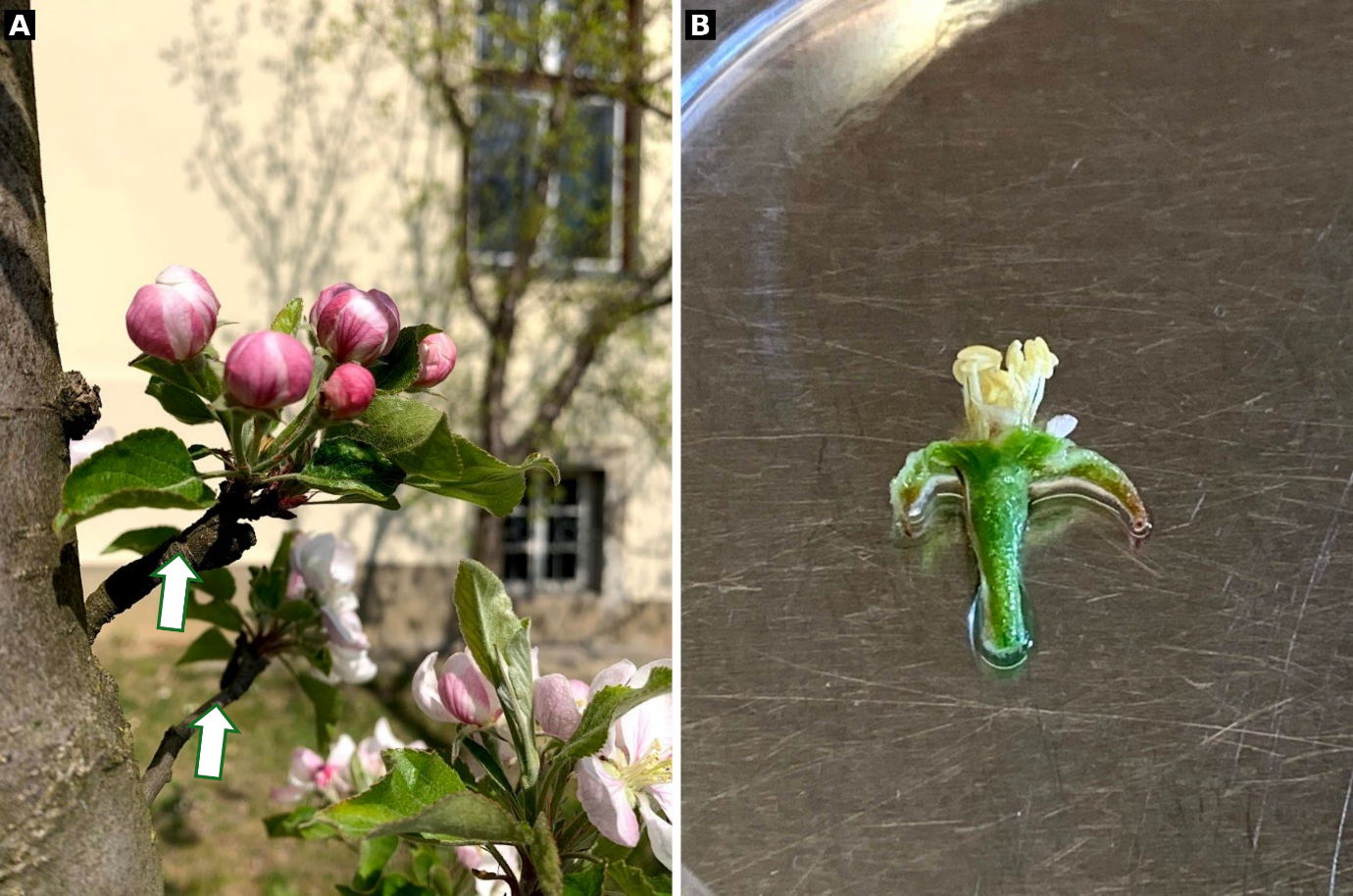


**Fig. S1.** Sampling of apple fruiting spurs (maternal vegetative woody tissue supporting the reproductive structures) before bloom. (A) Arrows denote the two fruiting spurs bearing flower buds before bloom. (B) Excised flower reproductive tissues (pollen and ovaries).

**
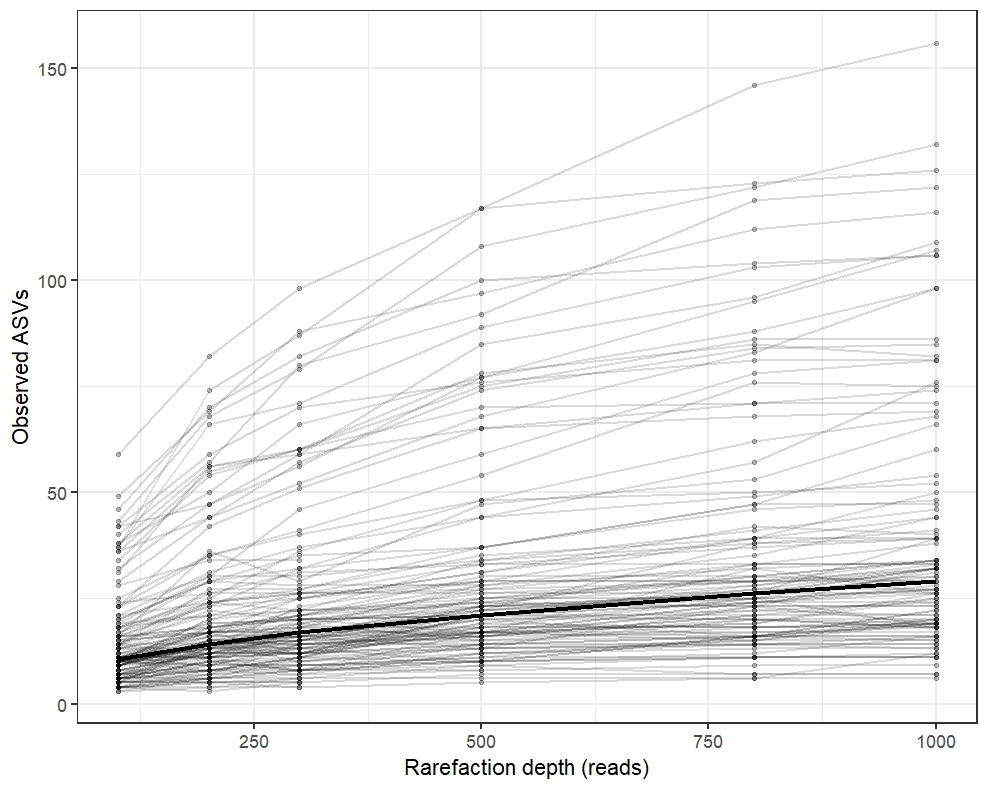
**

**Fig. S2.** Rarefaction curves by tissue type. Observed ASV richness was calculated after rarefying each sample to 100, 200, 300, 500, 800, and 1000 reads. Thin grey lines show individual samples; the thick black line shows the median across samples.

**
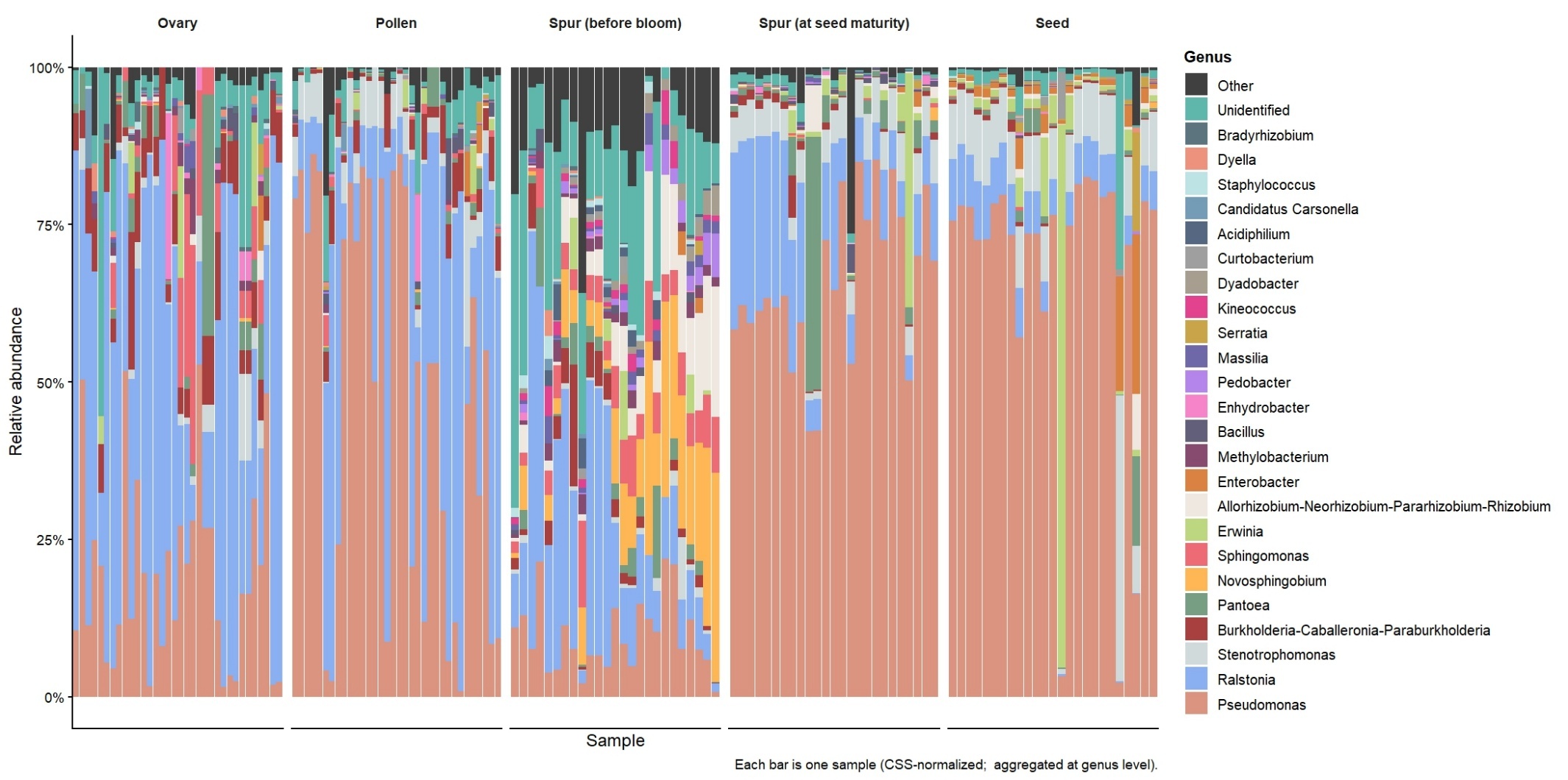
**

**Fig. S3**. Relative abundance of bacterial genera in ovary, pollen, spurs before bloom and spurs at seed maturity of the apple trees (*Malus domestica* BORKH. cv 'Gala Galaxy Selecta'). Stacked bar plots show the relative abundance of bacterial genera in each sample. Reads were CSS-normalized and agglomerated at the genus level. Genera shown explicitly correspond to the 24 most abundant genera across all samples plus “Unidentified” (including uncultured and unclassified ASVs); all remaining taxa are pooled as Other.


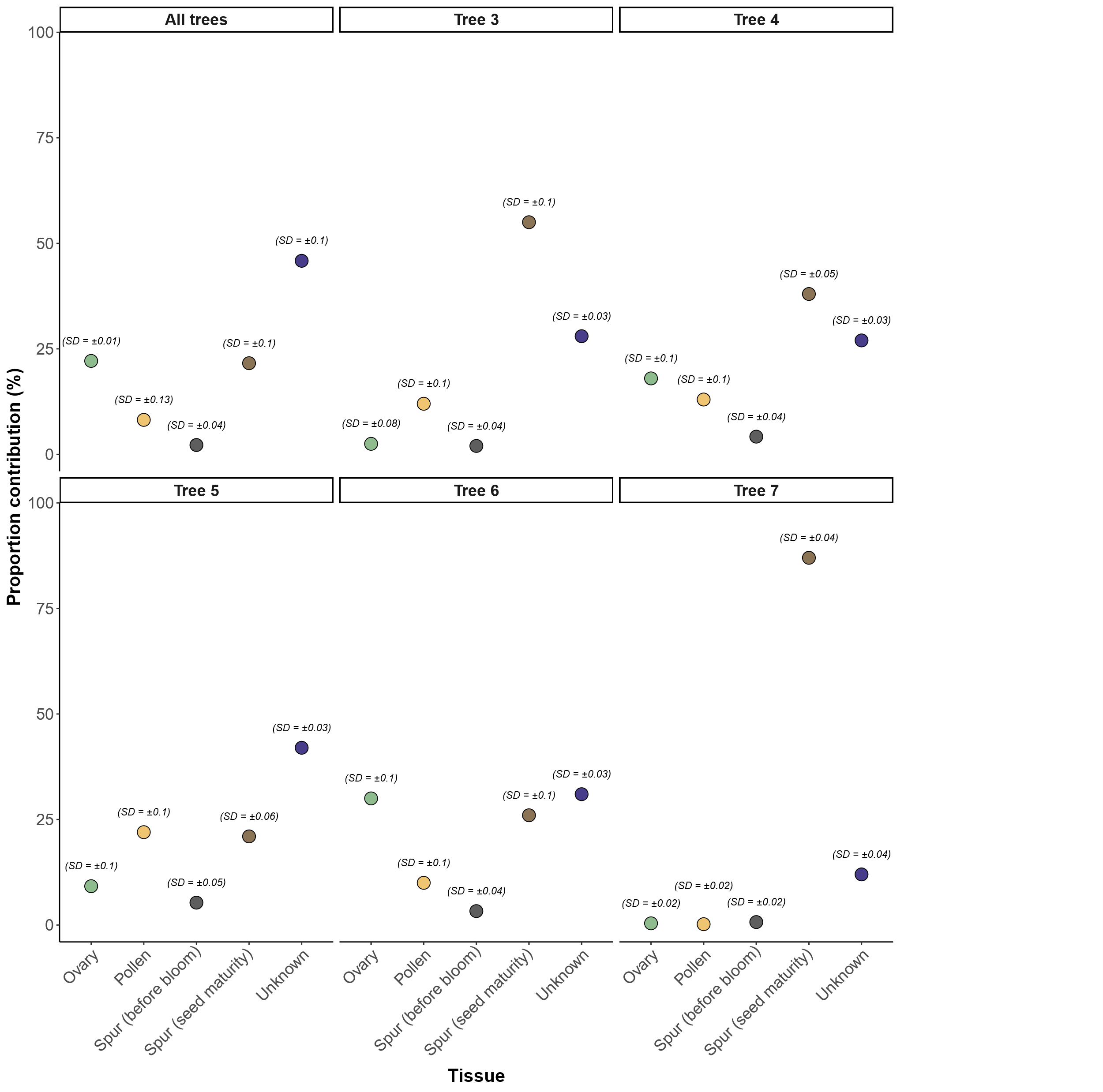


**Fig. S4**. Identification of potential asexual and sexual pathways of microbiome transmission to apple seeds using fast expectation-maximization for microbial source tracking (SourceTracker). Analyses were performed on ASV counts rarefied to 1,000 reads per tissue type. Circles represent mean proportion contributions from each tissue type, and the text above denotes the corresponding standard deviation (SD), which shows the model uncertainty. "Pooled Trees" refers to the aggregated results from all trees, while individual trees show the predicted contribution of each of tissue types to the seed microbiome for each of five trees. Unknown” represents the fraction not explained by sampled source tissues, e.g., ovary, pollen, spurs before bloom and at seed maturity.


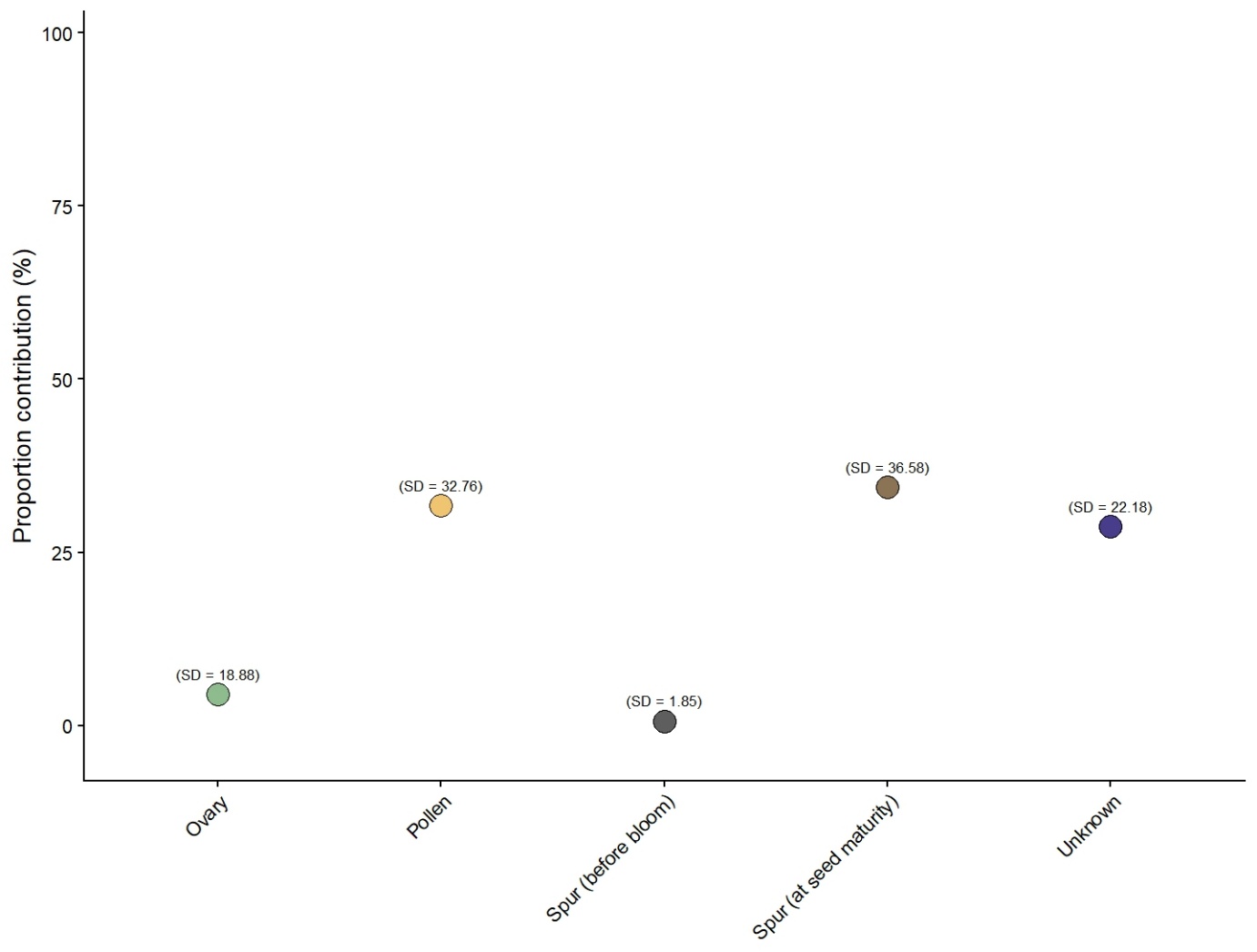


**Fig. S5**. Identification of potential asexual and sexual pathways of microbiome transmission to apple seeds using fast expectation-maximization for microbial source tracking (FEAST). Circles represent mean proportion contributions from each tissue type, and the text above denotes the corresponding standard deviation (SD), which depicts among-seed (sink) variability in estimated source contributions for each tissue type.

**
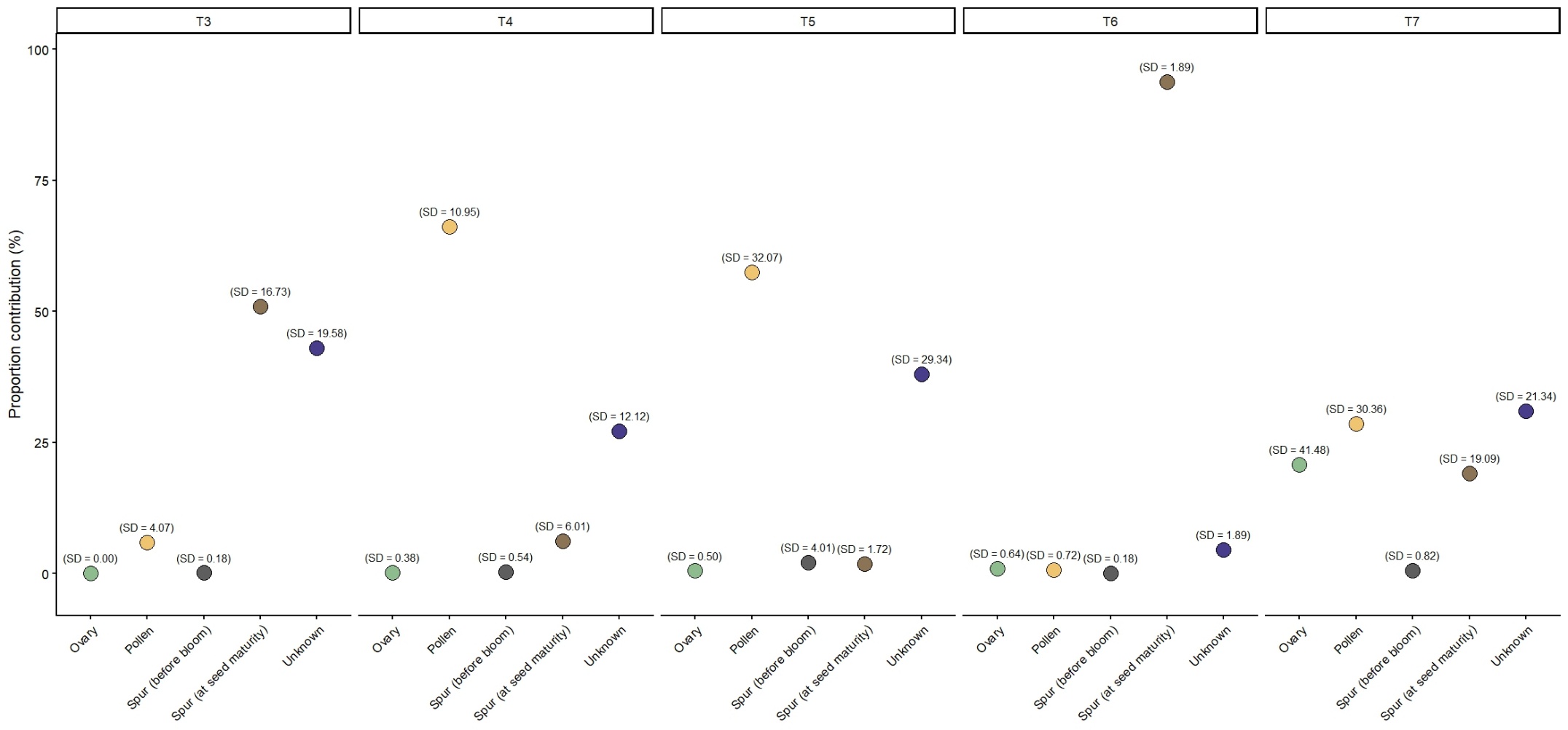
**

**Fig. S6**. Identification of potential asexual and sexual pathways of microbiome transmission to apple seeds using fast expectation-maximization for microbial source tracking (FEAST). (T3-T7) Show the predicted contribution of each of tissue types to seed microbiome for each of five sampled trees. Circles represent mean proportion contributions from each tissue type, and the text above denotes the corresponding standard deviation (SD), which depicts among-seed (sink) variability in estimated source contributions within each tree.
